# Mechanistic Insight into Tunable Spin Relaxation in Two-Dimensional Type-II Ligand-Perovskite Heterostructures

**DOI:** 10.1021/jacs.5c14647

**Published:** 2025-10-29

**Authors:** Ashish Soni, Cheng Yang, Yu-Ting Yang, Chenjian Lin, Letian Dou, Lili Wang

**Affiliations:** † Department of Chemistry, 1371 Emory University, Atlanta, Georgia 30322, United States; ‡ Davidson School of Chemical Engineering, 311308 Purdue University, West Lafayette, Indiana 47907, United States

## Abstract

Two-dimensional (2D) metal-halide perovskites with spin-dependent optical properties hold great promise for spintronic and quantum applications. However, their spin lifetimes, especially for *n* = 1 2D perovskites, are typically limited to subpicosecond time scales due to rapid spin relaxation driven by strong spin–orbit coupling (SOC), electron–hole exchange interactions, and phonon-mediated scattering. Here, we demonstrate that type-II ligand-perovskite heterostructures overcome these constraints by reducing electron–hole wave function overlap and exciton binding energy. Compared to the type-I 2D perovskite (PEA)_2_PbI_4_ with a spin lifetime of 0.29 ps at room temperature, our engineered type-II systems achieve substantially extended spin lifetimes, ∼6.37 ps for (4Tm)_2_PbI_4_ and ∼18.47 ps for (4TCNm)_2_PbI_4_. In both materials, spatial charge separation across the perovskite–ligand interface mitigates the Bir–Aronov–Pikus (BAP) mechanism. Temperature- and fluence-dependent measurements reveal Elliott–Yafet (EY)-dominated spin relaxation in (4Tm)_2_PbI_4_, consistent with the observation of coherent phonon oscillation, whereas (4TCNm)_2_PbI_4_ exhibits D’yakonov–Perel (DP)-dominated spin relaxation, with weaker phonon coupling further suppressing the EY relaxation, enabling spin lifetimes up to ∼126.81 ps at 5 K. Our findings establish a structural design framework for tailoring spin dynamics in 2D perovskites, offering a promising strategy to engineering spin and optoelectronic properties via rational ligand engineering.

## Graphical abstract

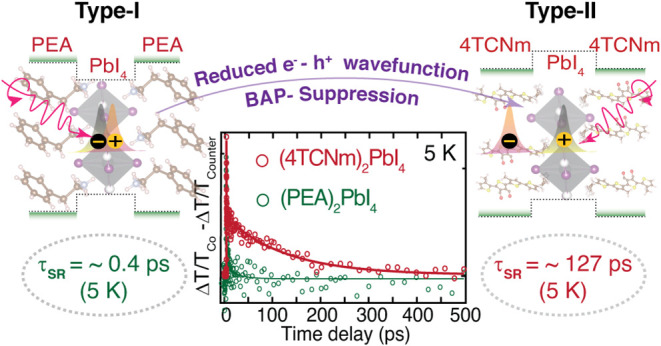

## Introduction

Among a broad family of spin-active materials, two-dimensional (2D) perovskites have garnered significant attention due to their structural tunability, ease of fabrication, and exceptional optoelectronic properties.^1–4^ However, their rapid spin relaxation remains a critical bottleneck for realizing practical spin-based devices. This limitation arises from the complex interplay of strong spin–orbit coupling, phonon scattering, and electron–hole exchange interactions, which together lead to ultrafast spin depolarization on subpicosecond time scales.^5–8^ Addressing this challenge requires a deeper understanding of the microscopic mechanisms governing spin relaxation and the ability to tailor them through materials design.

Traditional 2D perovskites with electronically insulating ligands form natural quantum wells with type-I band alignment, where quantum and dielectric confinement lead to tightly bound excitons localized within the inorganic layers.^9,10^ The resulting strong spatial overlap of carrier wave functions promotes ultrafast spin depolarization via electron–hole exchange interactions, a process described by the Bir–Aronov–Pikus (BAP) spin relaxation mechanism.^11^ In addition to BAP, spin relaxation can occur through the Elliott–Yafet (EY) and the D’yakonov–Perel (DP) mechanisms. In the EY mechanism, spin–orbit coupling induces mixing between spin-up and spin-down states, enabling spin flips during momentum scattering with phonons or impurities.^5,6^ In the DP mechanism,^12–14^ which occurs in materials lacking inversion symmetry, Rashba–Dresselhaus effects lift spin degeneracy, causing momentum-dependent spin precession that leads to depolarization during carrier scattering.

Spin relaxation dynamics have been extensively studied in type-I 2D perovskites.^15–18^ Qin et al.^5^ attributed the subpicosecond spin lifetimes in (MBA)_2_PbI_4_ to quantum confinement and reduced dielectric screening. In *n* = 1 2D perovskites, strong electron–hole wave function overlap favors the BAP mechanism as the dominant spin relaxation channel. To suppress BAP, previous studies have increased the layer thickness (*n*) in perovskites to reduce electron–hole exchange interactions. For instance, Song et al.^19^ observed BAP-dominated relaxation in *n* = 1–3 phenethylamine (PEA)-based perovskites, with a transition from BAP to EY for *n* > 3. Similarly, Chen et al. reported spin lifetimes increasing from ∼0.3 ps (*n* = 1) to ∼7 ps (*n* = 4) in 2D PEA-based perovskites at room temperature.^15^ Efforts to extend spin lifetimes in *n* = 1 2D perovskites have modulated the dielectric constant of insulating organic ligands. Chen et al.^20^ replaced butyl ammonium ligands with ethanol ammonium, which lowers the exciton binding energy and increases spin lifetime. Despite these efforts, reported spin lifetimes have remained limited to sub- to few-picosecond ranges,^21–24^ underscoring the need for alternative strategies and material architectures capable of supporting longer-lived spin states.

While most prior studies have focused on type-I 2D perovskites with electronically insulating ligands, type-II perovskites incorporating electronically active ligands offer a promising yet largely unexplored route to extend spin lifetimes. Their type-II band alignment facilitates ultrafast charge transfer between the inorganic perovskite layers and organic ligands, producing spatial charge separation that reduces electron–hole wave function overlap and holds promise for suppressing BAP relaxation.^25–27^ By enabling simultaneous control over carrier dynamics and electron–phonon coupling through ligand selection, type-II perovskites offer a powerful platform for probing spin relaxation pathways and advancing next-generation opto-spintronic materials.

Here, we report the temperature and fluence dependence of spin relaxation dynamics in type-I and type-II 2D perovskites using polarization-resolved transient absorption (TA) spectroscopy. We demonstrate a substantial enhancement in spin lifetimes—from 0.29 ps in (PEA)_2_PbI_4_ to 6.37 ps in (4Tm)_2_PbI_4_ and 18.47 ps in (4TCNm)_2_PbI_4_ at room temperature—accompanied by a transition in the dominant spin relaxation mechanism from BAP to EY and DP. Temperature scaling of the spin relaxation rate reveals EY-dominated relaxation in (4Tm)_2_PbI_4_ and DP-dominated relaxation in (4TCNm)_2_PbI_4_. This assignment is further supported by our coherent phonon measurements, which reveal weaker phonon coupling in (4TCNm)_2_PbI_4_, resulting in the longest spin lifetime among the three samples. Our findings provide a unified picture of how ligand engineering in 2D perovskites can tailor quantum well band alignments and lattice interactions, providing a mechanistic route to achieve targeted control over spin and carrier dynamics in low-dimensional semiconductors.

## Results and Discussion

### Exciton and Charge Transfer Dynamics

2D lead iodide hybrid perovskites were synthesized using three distinct organic ligands: phenethylammonium iodide (PEAI), 2-(3‴,4′-dimethyl-[2,2′:5′,2″:5″,2‴-quaterthiophen]-5-yl)­ethan-1-aminium iodide (4TmI), and 2-(3″,4″-dicyano-3‴,4′-dimethyl-[2,2′:5′,2″:5″,2‴-quaterthiophen]-5-yl)­ethan-1-aminium iodide (4TCNmI), as described in the Materials and Methods section. The chemical structures of these ligands are shown in Figure 1A. These ligands were selected to stabilize *n* = 1 2D Ruddlesden–Popper (RP) phase perovskites and to systematically tune the energy level offsets between the organic and inorganic components, thereby enabling control over interlayer charge transfer pathways. Band alignment diagrams for these specific 2D perovskite heterostructures have been reported previously,^25,26^ and are summarized in Figure 1B. In (4Tm)_2_PbI_4_, the band alignment favors hole transfer from the valence band maximum (VBM) of the inorganic layer to the highest occupied molecular orbital (HOMO) of the 4Tm ligand. In (4TCNm)_2_PbI_4_, electron transfer from the conduction band minimum (CBM) of the inorganic layer to the lowest unoccupied molecular orbital (LUMO) of the 4TCNm ligand is energetically favorable. These alignments define both systems as type-II heterostructures, where interlayer charge separation is expected to occur across the organic–inorganic interface.

**Figure 1:**
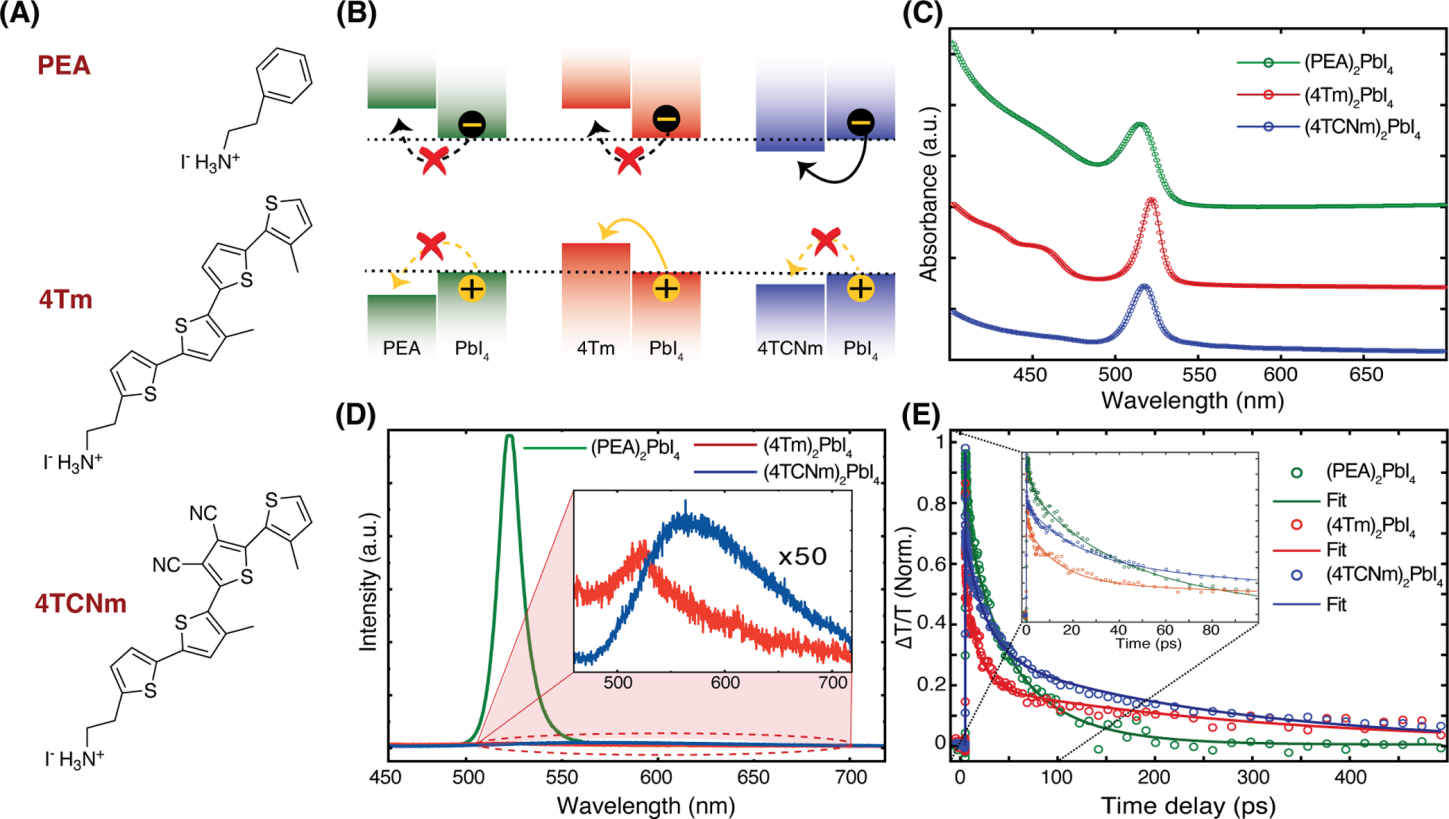
Sample characterization and band alignment of 2D perovskites with organic ligands. (A) Chemical structures of the organic ligands (PEAI, 4TmI, and 4TCNmI) used to prepare 2D perovskite thin films. (B) Schematic illustration of energy band alignment between the organic and inorganic layers of perovskite heterostructures. Solid arrows indicate allowed interlayer charge transfer, while dashed arrows with a cross mark represent energetically unfavorable transitions. (C) Linear absorption spectra of (PEA)_2_PbI_4_, (4Tm)_2_PbI_4_, and (4TCNm)_2_PbI_4_. (D) Corresponding steady-state photoluminescence (PL) spectra. Significant PL quenching is observed in type-II (4Tm)_2_PbI_4_, and (4TCNm)_2_PbI perovskites, shown in the inset with 50× magnification. (E) Transient absorption (TA) decay kinetics probed at the excitonic transitions of (PEA)_2_PbI_4_ (518 nm), (4Tm)_2_PbI_4_ (520 nm), and (4TCNm)_2_PbI_4_ (516 nm), under 500 nm pump excitation at a fluence of 1.75 μJ/cm^2^.

To evaluate the influence of band offsets on exciton dynamics, we measured the linear absorption spectra for all three samples (Figure 1C), each showing a prominent excitonic feature near 515–520 nm. We extracted exciton binding energies by fitting the spectra using the 2D Elliot’s model^20,28^ (Figure S1). The exciton binding energy of (PEA)_2_PbI_4_ exceeds 400 meV, whereas both (4Tm)_2_PbI_4_ and (4TCNm)_2_PbI_4_ exhibit substantially reduced binding energies (<60 meV). This decrease indicates weakened Coulomb interactions and enhanced dielectric screening, consistent with interfacial charge separation in the type-II systems.^26,29–31^ These findings align with the steady-state photoluminescence (PL) spectra shown in Figure 1D. (PEA)_2_PbI_4_ displays strong PL emission, which is characteristic of efficient radiative recombination of tightly bound excitons. In contrast, (4Tm)_2_PbI_4_ and (4TCNm)_2_PbI_4_ exhibit markedly quenched PL (inset of Figure 1D), supporting photoinduced charge transfer across the organic–inorganic interface.

To further probe the charge transfer dynamics, we performed transient absorption (TA) spectroscopy using a linearly polarized pump (500 nm, 1.75 μJ/cm^2^ fluence) and probe beams. The decay kinetics of all three samples, probed at their respective excitonic transitions, are compared in Figure 1E. (PEA)_2_PbI_4_ exhibits dynamics consistent with exciton recombination in type-I systems.^25,32^ In contrast, both (4Tm)_2_PbI_4_ and (4TCNm)_2_PbI_4_ show pronounced early time quenching (inset of Figure 1E, with kinetic fits in Table S1), arising from interlayer charge transfer enabled by their type-II band alignment. Specifically, hole transfer in (4Tm)_2_PbI_4_ occurs on an ∼13 ps time scale, while electron transfer in (4TCNm)_2_PbI_4_ occurs on an ∼17 ps time scale. These values are in good agreement with previously reported charge transfer time scales in these 2D perovskite heterostructures.^25^ Additionally, a long-lived component (τ > 500 ps) is observed in both type-II systems, likely arising from persistent bleaching of the excitonic transition due to interlayer charge separation. The corresponding interlayer carrier recombination has been reported to occur on nanosecond time scales,^25^ beyond the temporal window of our measurements.

### Ultrafast Spin Dynamics Probed by Circularly Polarized Transient Absorption

Building on our understanding of interlayer charge separation in these type-II perovskite heterostructures, we next investigated how these heterostructures influence spin relaxation dynamics. To this end, we employed spin-resolved TA spectroscopy using a circularly polarized pump tuned near excitonic resonance (500 nm) and a circularly polarized broadband probe, as discussed in the Materials and Methods section. The band structure of lead halide perovskites yields a simple two-level manifold at the band edge that is double degenerate.^33–36^ Optical selection rules^20^ dictate that right (σ^+^) and left (σ^–^) circularly polarized light selectively excite excitons with in |+1⟩ and |−1⟩ spin states, respectively (inset of Figure 2A). By probing with circularly polarized light of the same (co-circular) or opposite (counter-circular) helicity as the pump, transient absorption spectroscopy tracks the population imbalance and relaxation between these spin-polarized excitonic states. Monitoring the temporal evolution of these signals reveals the spin relaxation dynamics of the material.

**Figure 2:**
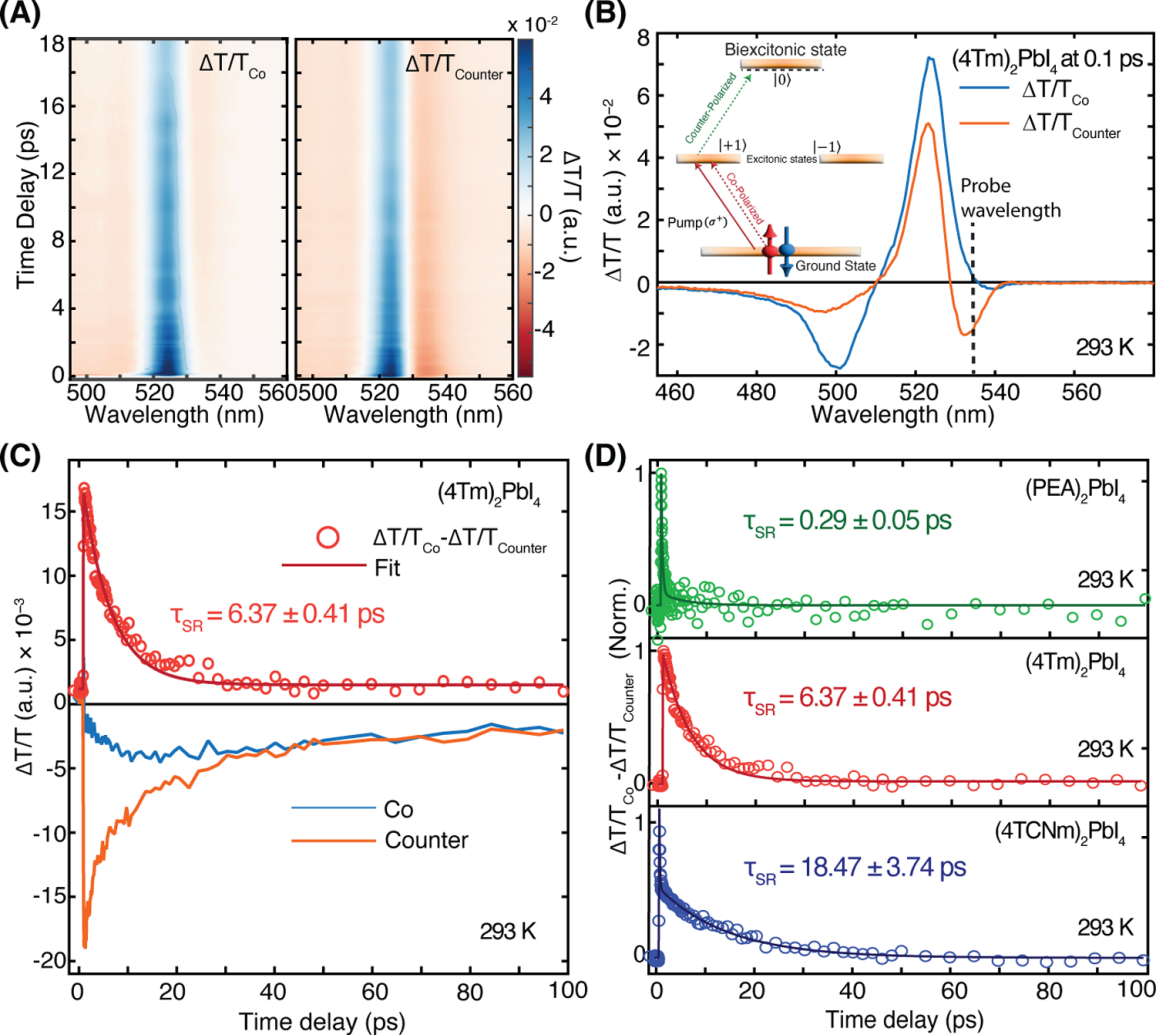
Circularly polarized TA spectra and spin relaxation dynamics at room temperature. (A) 2D TA contour plots of (4Tm)_2_PbI_4_ under co- and counter-circularly polarized pump–probe configurations with near-resonance excitation at 500 nm and a fluence of 3.5 μJ/cm^2^. (B) TA spectra of (4Tm)_2_PbI_4_ at 0.1 ps delay, comparing co- and counter-polarized configurations. Inset: Schematic illustration of spin-dependent biexciton formation, in which counter-circular polarized excitation promotes the exciton into a biexciton state, giving rise to the negative photoinduced absorption (PIA) feature around 530 nm. (C) TA kinetics of (4Tm)_2_PbI_4_ at 535 nm under co- and counter-polarized configurations. The difference signal (Δ*T*/*T*_Co_ – Δ*T*/*T*_Counter_), shown in red, reflects spin relaxation dynamics and is fit with a single-exponential function to extract the spin relaxation time (τ_SR_) at 293 K. (D) Comparison of normalized spin relaxation dynamics (Δ*T*/*T*_Co_ – Δ*T*/*T*_Counter_) for (PEA)_2_PbI_4_, (4Tm)_2_PbI_4_, and (4TCNm)_2_PbI_4_ at 293 K. Solid lines represent exponential fits to extract τ_SR_ for each sample.

The 2D TA contour plot of (4Tm)_2_PbI_4_ (Figure 2A) reveals distinct spectral responses under co- and counter-circularly polarized pump–probe configurations. A prominent PIA feature, centered around 535 nm, is clearly visible under the counter-polarized configuration but is significantly weaker in the copolarized case. This polarization-dependent feature is further resolved in Figure 2B, which shows the spectral profiles at 0.1 ps for both configurations. This PIA signal is attributed to biexciton formation (inset of Figure 2B), in agreement with prior reports demonstrating that spin-selective biexciton generation occurs only between excitons with opposite spins.^20^ Similar spin-dependent PIA features are observed in both (PEA)_2_PbI_4_ and (4TCNm)_2_PbI_4_, with their TA contour plots and spectral profiles shown in Supporting Figure S3.

To quantify spin relaxation dynamics in (4Tm)_2_PbI_4_, we analyzed TA kinetics at 535 nm under co- and counter-circularly polarized pump–probe configurations (Figure 2C, blue and orange traces). The difference between these traces (Δ*T*/*T*_Co_ – Δ*T*/*T*_Counter_), shown as red circles, reflects the decay of spin-polarized states to equilibrium. Fitting this difference signal with a single-exponential decay yields a spin relaxation time (τ_SR_) of ∼6.37 ps in (4Tm)_2_PbI_4_. We performed similar analyses for (PEA)_2_PbI_4_ and (4TCNm)_2_PbI_4_ (Supporting Figure S4) and compared the extracted τ_SR_ for all three samples (Figure 2D). At room temperature, type II perovskites (4Tm)_2_PbI_4_ and (4TCNm)_2_PbI_4_ exhibit τ_SR_ of ∼6.37 and ∼18.47 ps, respectively, both dramatically longer than ∼0.29 ps observed for the type I reference (PEA)_2_PbI_4_. The substantial enhancement of τ_SR_ in type-II perovskite heterostructures likely arises from spatial charge separation across the organic–inorganic interface, which suppresses spin depolarization by weakening electron–hole exchange interactions and exciton binding. These results highlight interfacial band engineering as a promising approach for extending spin lifetimes in 2D perovskites—an essential step toward their integration into spintronic and quantum optoelectronic applications.

### Mechanistic Origins of Spin Relaxation: Distinguishing BAP, EY, and DP Pathways

The significant variation in spin lifetimes among these samples suggests distinct underlying spin relaxation pathways. In addition to the contrast between type-I and type-II perovskites, we find that (4Tm)_2_PbI_4_ and (4TCNm)_2_PbI_4_, which differ only in two cyano substituents on the ligand backbone, exhibit markedly different spin dynamics. This divergence underscores the remarkable sensitivity of spin relaxation to subtle variations in ligand structure and electronic coupling. To elucidate the dominant spin relaxation mechanisms, we systematically investigated the temperature and excitation fluence dependence of spin relaxation across these systems.

In 2D perovskites, three primary mechanisms have been proposed for spin depolarization: Elliott–Yafet (EY), D’yakonov–Perel (DP), and Bir–Aronov–Pikus (BAP) mechanisms.^20,37^ These mechanisms differ fundamentally in how spin couples with scattering processes or the carrier interactions and can therefore be distinguished through their characteristic temperature and excitation fluence dependence. The EY mechanism is intrinsic to the material with SOC, where spin relaxation arises via spin-flip processes during momentum scattering with phonons or lattice impurity, resulting in strong temperature dependence.^20,38^ In contrast, the DP mechanism occurs in materials lacking inversion symmetry, where spin degeneracy is lifted at nonzero wavevector due to Rashba–Dresselhaus type spin splitting. The resulting momentum-dependent effective magnetic field induces spin precession, ultimately leading to spin relaxation with random momentum scattering events.^39,40^ The DP mechanism typically shows a weaker temperature dependence than EY. Finally, the BAP mechanism arises from electron–hole exchange interactions, where the spin of an electron can flip through coupling with the spin of a hole and vice versa. BAP-driven spin relaxation depends critically on the exchange interaction strength, which is governed by the overlap of electron and hole wave functions. This mechanism is particularly relevant in 2D perovskites, where strong dielectric and quantum confinement give rise to tightly bound excitons.^17^ Unlike the EY and DP mechanisms, BAP-driven spin relaxation is insensitive to temperature, but its rate scales linearly with carrier density and can thus be probed by varying the excitation fluence.

As the temperature decreased to 5 K, spin relaxation dynamics differ dramatically across the three samples: the type-I reference (PEA)_2_PbI_4_ shows negligible temperature dependence, with τ_SR_ remaining ∼0.39 ps at 5 K, whereas the type II perovskites (4Tm)_2_PbI_4_ and (4TCNm)_2_PbI_4_ exhibit markedly extended spin relaxation times of ∼39 and ∼127 ps, respectively (Supporting Figure S5). Representative temperature-dependent spin relaxation dynamics for (4Tm)_2_PbI_4_ are shown in Figure 3A, where τ_SR_ increases from ∼6 ps at 293 K to 39 ps at 5 K. Similar temperature-dependent measurements for (PEA)_2_PbI_4_ and (4TCNm)_2_PbI_4_ are provided in Supporting Figure S6.

**Figure 3:**
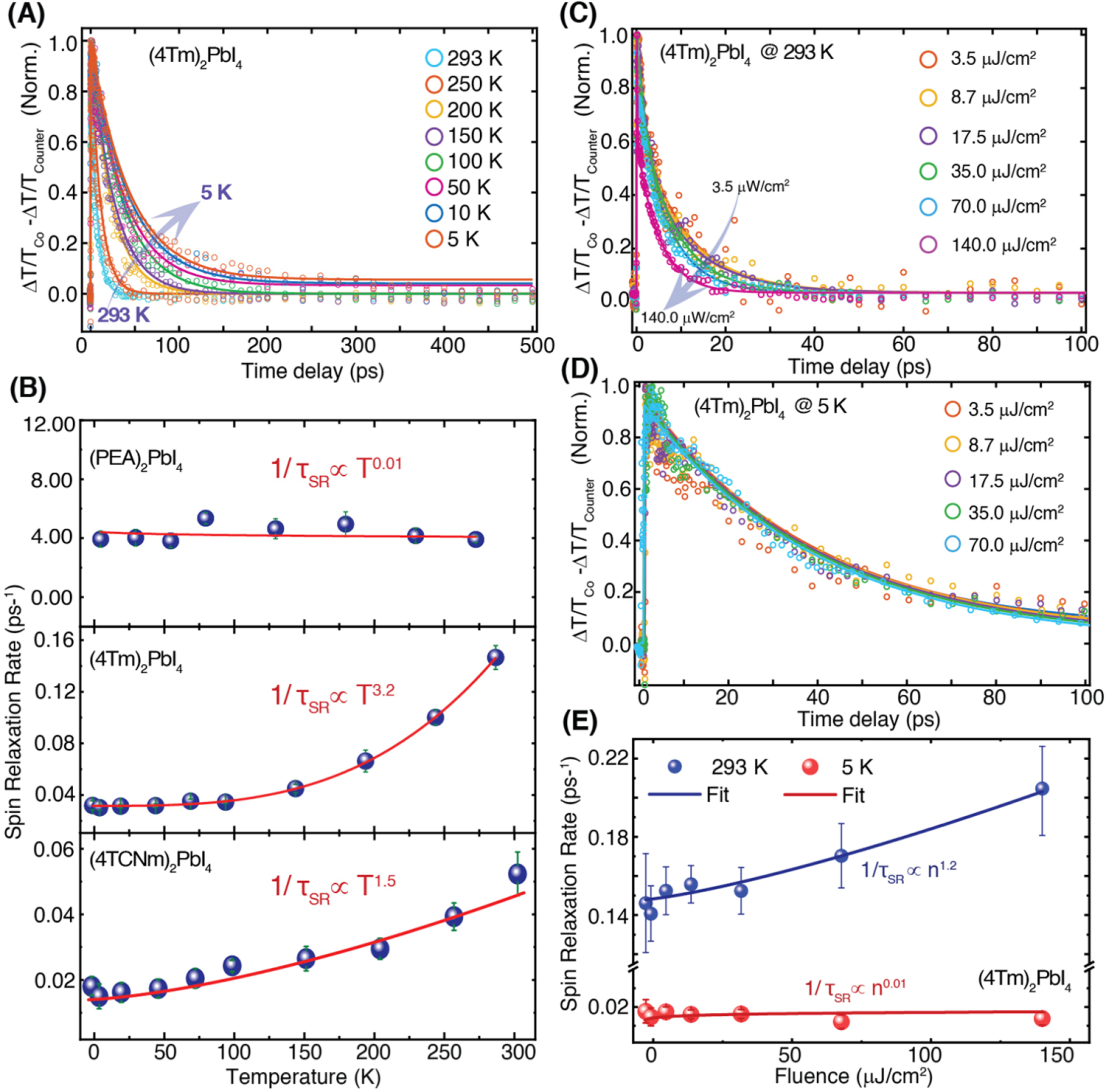
Temperature and excitation fluence-dependent spin relaxation rate. (A) Spin relaxation dynamics (Δ*T*/*T*_Co_ – Δ*T*/*T*_Counter_) for (4Tm)_2_PbI_4_ at an excitation fluence of 3.5 μJ/cm^2^ and different temperatures. (B) Temperature dependence of the spin relaxation rate (1/τ_SR_) for (PEA)_2_PbI_4_, (4Tm)_2_PbI_4_, and (4TCNm)_2_PbI_4_ from 293 to 5 K. (PEA)_2_PbI_4_ shows negligible temperature dependence, whereas (4Tm)_2_PbI_4_ and (4TCNm)_2_PbI_4_ exhibit temperature scaling of *T* ^3.2^ and *T* ^1.5^, respectively. (C, D) Normalized spin relaxation dynamics for (4Tm)_2_PbI_4_ at varying excitation fluences and at (C) 293 K and (D) 5 K. (E) Excitation fluence dependence of spin relaxation rate for (4Tm)_2_PbI_4_ at 293 and 5 K. The spin relaxation rate increases nearly linearly with fluence at 293 K but is fluence-independent at 5 K.

To elucidate the temperature dependence of spin relaxation, we extracted the spin relaxation rates (γ_SR_ = 1/τ_SR_) of all three samples. We plotted them as a function of temperature, fitting the data to a power-law^38^ relationship of γ_SR_ ∝ *T* ^b^ (Figure 3B). For type-I reference (PEA)_2_PbI_4_, γ_SR_ remains nearly constant (γ_SR_ ∝ *T* ^0.01^) across the 5–293 K range. This temperature insensitivity is characteristic of the BAP mechanism. While other pathways such as DP and EY may still contribute, the absence of observable temperature dependence demonstrates the critical role of BAP-driven spin relaxation in (PEA)_2_PbI_4_.^41^ By contrast, the type-II perovskites show markedly different behavior. (4Tm)_2_PbI_4_ exhibits a steep temperature dependence of spin relaxation, with γ_SR_ ∝ *T* ^3.2^, while (4TCNm)_2_PbI_4_ shows a weaker but still appreciable scaling of γ_SR_ ∝ *T* ^1.5^. Despite their structural similarity, the observed disparity in temperature dependence suggests distinct underlying spin relaxation mechanisms, likely arising from differences in electron–phonon coupling strengths between the two samples. The strong *T* ^3.2^ dependence in (4Tm)_2_PbI_4_ indicates phonon-mediated spin relaxation, consistent with the EY mechanism. In the EY model, spin relaxation occurs through phonon-assisted momentum scattering events, and the spin relaxation rate γ_SR_^EY^ is given by^42,43^

1 $$\gamma_{\mathrm{SR}}^{\mathrm{EY}}=A{\left(\frac{k_{B}}{E_{g}}\right)}^{2}\eta^{2}{\left(\frac{1-\eta /2}{1-\eta /3}\right)}^{2}\frac{1}{\tau_{p}}\times T^{2}$$

Here, *E*_g_ is the bandgap, η = Δ/(*E*_g_ + Δ), Δ is the spin–orbit splitting of the valence band, and τ_p_ is the phonon scattering time, which scales with temperature as τ_p_^–1^ ∝ *T* ^3/2^.^42^ The resulting overall scaling of γ_SR_^EY^ ∝ *T* ^7/2^ agrees well with our experimental observation in (4Tm)_2_PbI_4_. For (4TCNm)_2_PbI_4_, however, the weaker temperature dependence (γ_SR_ ∝ *T* ^1.5^) aligns more closely with the DP mechanism. In this model, spin relaxation arises from spin precession induced by Rashba–Dresselhaus spin splitting in noncentrosymmetric crystals, and the spin relaxation rate is given by^43^

2 $$\gamma_{\mathrm{SR}}^{\mathrm{DP}}=Q\alpha^{2}\frac{{k_{B}}^{3}}{\hbar^{2}E_{g}}\tau_{p}\times T^{3}$$

where *Q* is a dimensionless constant, and α is related to the effective mass of conduction electrons and the electron rest mass. Given that the phonon scattering rate scales as τ_p_^–1^ ∝ *T* ^3/2^, the overall DP-driven spin relaxation rate is expected to follow the order γ_SR_^DP^ ∝ *T* ^3/2^, in good alignment with our experimental observation for (4TCNm)_2_PbI_4_.

The distinct temperature scaling trends observed across these samples highlight the complex interplay of multiple spin relaxation pathways. In type-I perovskite (PEA)_2_PbI_4_, BAP is the primary relaxation pathway. In contrast, type-II systems exhibit spatial charge separation at the organic–inorganic interface, which weakens electron–hole exchange interactions, thereby suppressing BAP contributions and shifting the balance toward other relaxation pathways. Within type-II (4Tm)_2_PbI_4_ and (4TCNm)_2_PbI_4_, the prevailing spin relaxation mechanism, whether EY or DP, further depends on the strength of spin–phonon coupling, which is modulated by ligand structure and crystal lattice asymmetry. To further probe this mechanistic framework and specifically assess the role of BAP, we examined the fluence dependence of spin relaxation in all three samples. Representative normalized spin dynamics of (4Tm)_2_PbI_4_ under increasing excitation fluences are shown at 293 and 5 K in Figure 3C,D, respectively. At 293 K, spin relaxation accelerates with increasing fluence, indicating the presence of exchange-driven BAP contribution that scales with carrier density. In contrast, spin relaxation remains effectively fluence-independent at 5 K, with fitted spin lifetimes unchanged across a fluence range of 3.5–70 μJ/cm^2^. Minor deviations at early times in the overlaid traces in Figure 3D stem from coherent phonon oscillations (CPO) in (4Tm)_2_PbI_4_, which are discussed in the following section. This trend is quantitatively captured in Figure 3E, where the spin relaxation rate (γ_SR_) is plotted as a function of fluence at both temperatures. At 293 K, γ_SR_ exhibits near-linear scaling with carrier densities (γ_SR_ ∝ *n* ^1.2^), whereas at 5 K, it remains almost constant (γ_SR_ ∝ *n* ^0.01^). This lack of fluence dependence suggests near-complete suppression of the BAP contribution at low temperatures. Taken together with the strong temperature scaling of γ_SR_ ∝ *T* ^3.2^, these results establish phonon-mediated EY relaxation as the dominant mechanism in (4Tm)_2_PbI_4_, with only minor BAP contributions persisting at room temperature, likely due to slightly enhanced electron–hole exchange interactions at elevated temperatures.

In contrast to (4Tm)_2_PbI_4_, both (PEA)_2_PbI_4_ and (4TCNm)_2_PbI_4_ exhibit clear fluence-dependent spin relaxation at both room and cryogenic temperatures (Figures S7 and S8). While the extended spin lifetimes in (4TCNm)_2_PbI_4_ (∼18 ps at 293 K and ∼127 ps at 5 K) suggest partial suppression of BAP relaxation, the persistence of fluence dependence indicates that BAP remains a non-negligible contributor. This residual BAP contribution is likely due to less effective and slower interlayer charge separation compared to (4Tm)_2_PbI_4_ (Figure 1E).^43,44^ When considered alongside the moderate temperature dependence of γ_SR_ ∝ *T* ^1.5^, these results point to a dominant DP spin relaxation in (4TCNm)_2_PbI_4_, with an appreciable residual BAP contribution. In contrast, the persistent fluence dependence and temperature-insensitive spin relaxation rate in the type-I reference (PEA)_2_PbI_4_ further support BAP as the primary relaxation pathway, although minor contributions from EY or DP mechanisms cannot be excluded.

### Coherent Phonon Oscillations

Interestingly, although (4TCNm)_2_PbI_4_ exhibits less complete suppression of BAP relaxation compared to (4Tm)_2_PbI_4_, it demonstrates a significantly longer spin lifetime (∼18 vs 6 ps at 293 K) despite the structural similarity of their ligands. This nearly 3-fold difference and their distinct temperature dependence suggest that an additional factor beyond BAP suppression, such as differences in phonon coupling strength, may influence spin relaxation. To further investigate the role of phonon interactions in modulating spin dynamics, we examined coherent phonon oscillations (CPO) in all three samples. In 2D perovskites, strong exciton–phonon coupling can give rise to CPO, where periodic lattice vibrations coherently modulate the materials’ optical susceptibility.^45–47^ These oscillations can be sensitively captured by transient absorption spectroscopy, providing a direct, time-resolved probe of the lattice dynamics. At 5 K, upon near-resonant excitation with linear polarized pump, both (PEA)_2_PbI_4_ and (4Tm)_2_PbI_4_ exhibit pronounced oscillatory features superimposed on their exponential decay profiles (top two panels, Figure 4A), a signature of coherent phonon oscillation. In stark contrast, (4TCNm)_2_PbI_4_ displays no discernible oscillations (bottom panel, Figure 4A), suggesting significantly weaker phonon coupling, relative to the other two samples. To isolate these oscillatory components, we subtracted the fitted exponential decay background from each kinetic trace. The resulting oscillatory residuals, shown in Figure 4B, were fit to a damped cosine function to extract the phonon frequencies and dephasing time. The fitting function used is

3 $$I\left(t\right)=\sum_{n=1}^{i}A_{i}\;\cos\left(2\pi f_{i}\left(t-t_{0}\right)+\phi_{i}\right)e^{-t/\tau_{i}}$$

where *A*_*i*_ is the oscillation amplitude, *f*_*i*_ is the phonon frequency, ϕ_*i*_ is the phase shift, and τ_*i*_ is the damping time. Temperature-dependent CPO data and the corresponding fits are provided in Supporting Figure S9, showing a decrease in oscillation amplitude and dephasing time as temperature increases.

**Figure 4:**
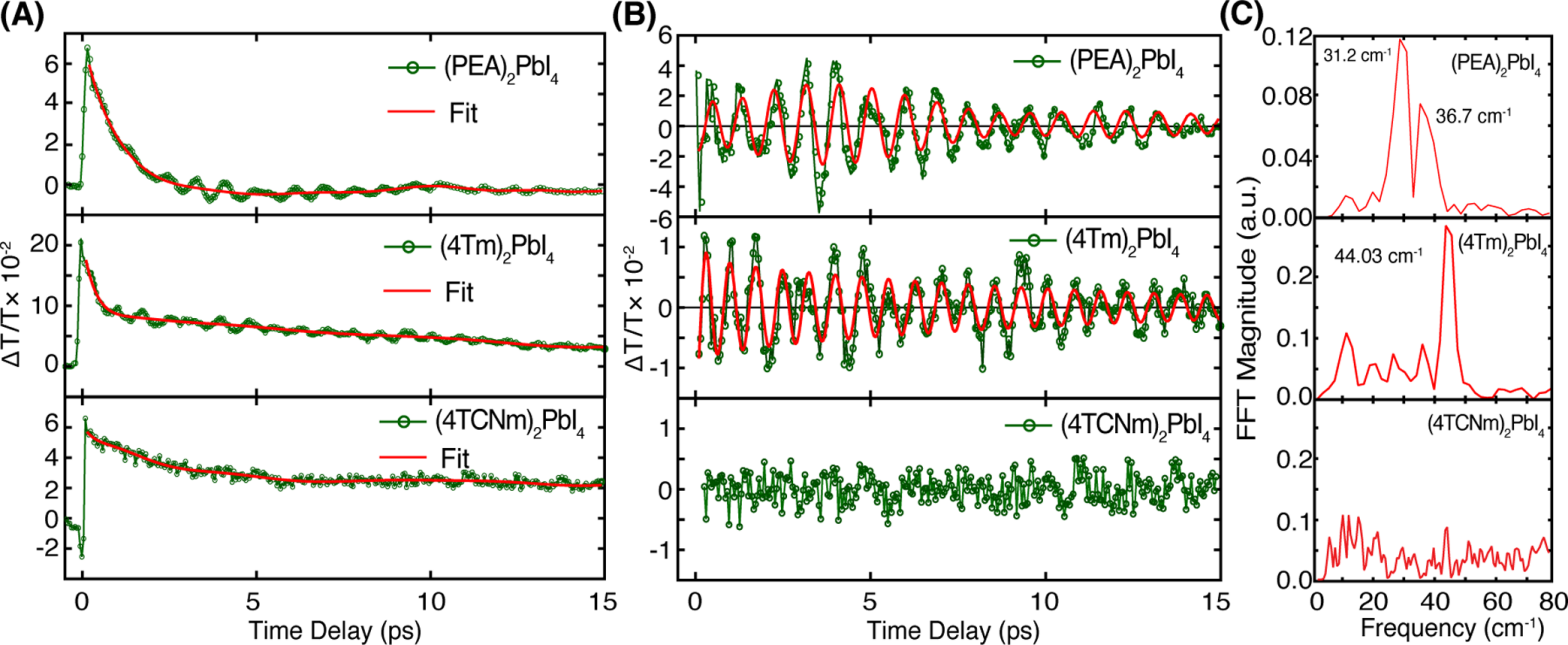
Coherent phonon oscillations at 5 K. (A) Transient absorption (TA) kinetic traces measured with linearly polarized pump and probe. Pronounced coherent phonon oscillations (CPO) are observed in (PEA)_2_PbI_4_ and (4Tm)_2_PbI_4_, but not (4TCNm)_2_PbI_4_. (B) Residual oscillatory signal obtained after subtracting the exponential decay from the TA kinetics for (PEA)_2_PbI_4_, (4Tm)_2_PbI_4_, and (4TCNm)_2_PbI_4_. (C) Fast Fourier transform (FFT) analysis of the residual oscillations in panel (B). (4TCNm)_2_PbI_4_ exhibits no discernible coherent phonon modes.

The corresponding fast Fourier transform (FFT) analysis of these samples further confirms the presence of low-frequency phonon modes (Figure 4C). Specifically, (PEA)_2_PbI_4_ exhibits characteristic frequencies at around ∼31.2 and ∼36.7 cm^–1^, and (4Tm)_2_PbI_4_ shows a dominant mode near ∼44.3 cm^–1^. These features are assigned to the Pb–I–Pb bending and octahedral twist modes of the inorganic network.^46,48^ In contrast, coherent phonon modes are absent in the FFT analysis of (4TCNm)_2_PbI_4_, reinforcing the interpretation of significantly weaker phonon coupling in this system. The pronounced CPO observed in (4Tm)_2_PbI_4_ indicates robust phonon coupling, which can facilitate EY-driven spin relaxation mediated by phonon-assisted momentum scattering. The weaker phonon coupling in (4TCNm)_2_PbI_4_ suggests the possible suppression of EY relaxation in this sample, which aligns with its longer spin lifetimes and weaker temperature dependence (γ_SR_ ∝ *T* ^1.5^), supporting our assignment of DP-dominated spin relaxation.

To summarize the mechanistic insights derived from our transient spin dynamics and coherent phonon measurements, we present a schematic overview of the proposed relaxation pathways in these samples (Figure 5). In the type-I (PEA)_2_PbI_4_, excitons remain tightly confined within the inorganic layer, resulting in strong electron–hole wave function overlap that facilitates ultrafast spin relaxation (∼0.3 ps at both 5 and 293 K), primarily via the BAP mechanism. Additional minor contributions from EY pathways are also possible, as suggested by the pronounced coherent phonon oscillations observed in this sample. In type-II (4Tm)_2_PbI_4_, hole transfer to the organic ligand layer leads to spatial charge separation, which effectively suppresses the BAP relaxation and prolongs spin lifetimes to ∼6.37 ps at 293 K and ∼39.14 ps at 5 K (Table 1). (4Tm)_2_PbI_4_ also exhibits strong coherent phonon oscillations, signatures of strong electron–phonon coupling. Together with the steep temperature dependence of the spin relaxation rate (γ_SR_ ∝ *T* ^3.2^), these observations support EY-mediated relaxation as the dominant pathway in (4Tm)_2_PbI_4_. In contrast, (4TCNm)_2_PbI_4_ displays even longer spin lifetimes (∼18.47 ps at 293 K and ∼126.81 ps at 5 K, see Table 1), despite less effective quenching of BAP relaxation compared to (4Tm)_2_PbI_4_. This enhancement is attributed to significantly weaker phonon couplings, as evidenced by the absence of detectable coherent phonon oscillations, and points to a shift toward DP-dominated spin relaxation with residual BAP contributions.

**Figure 5:**
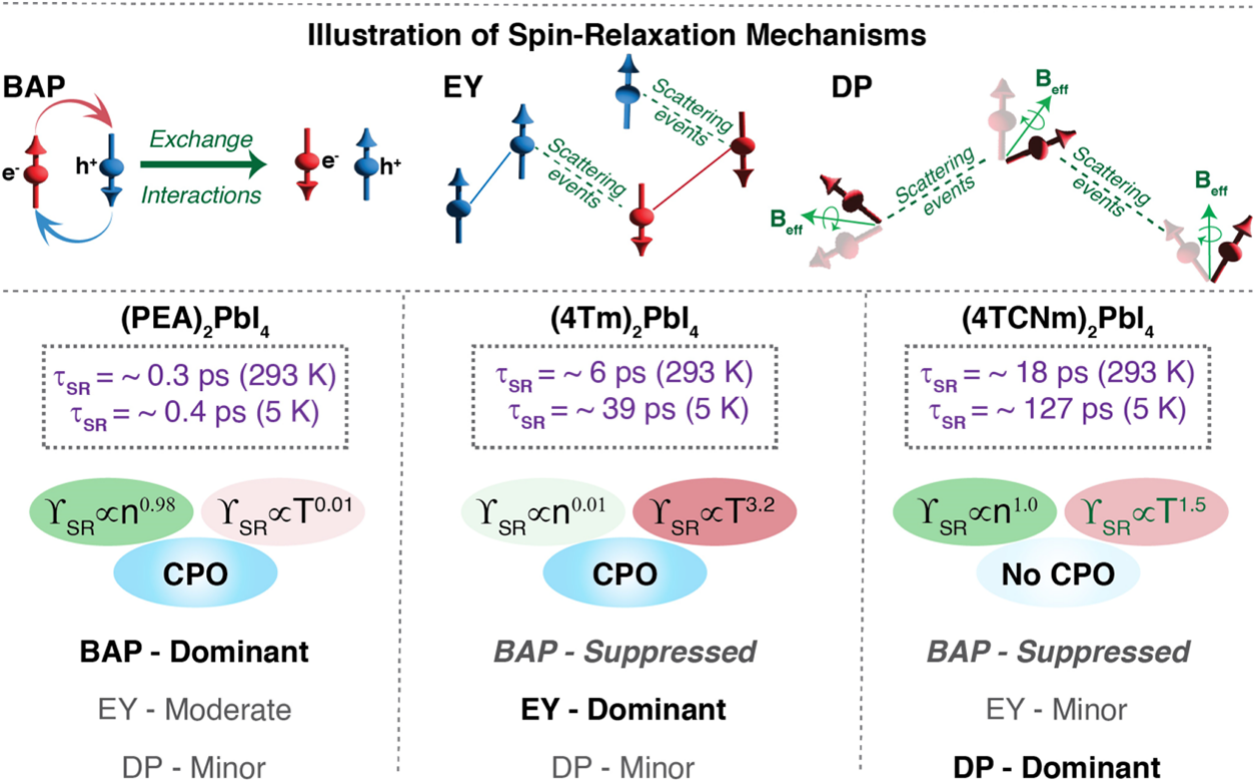
Schematic representation of major spin relaxation mechanisms in (PEA)_2_PbI_4_ (type-I), (4Tm)_2_PbI_4_ (type-II), and (4TCNm)_2_PbI_4_ (type-II).

**Table 1:** Spin Relaxation Time for 2D Ligand–Perovskite Heterostructures at 293 and 5 K

		relaxation time (τ_SR_) (ps)	
sample	band alignment	293 K	5 K
(PEA)_2_PbI_4_	type-I (no charge transfer)	0.29 ± 0.05	0.39 ± 0.09
(4Tm)_2_PbI_4_	type-II (hole transfer)	6.37 ± 0.41	39.14 ± 2.43
(4TCNm)_2_PbI_4_	type-II (electron transfer)	18.47 ± 3.74	126.81 ± 25.76

Together, these findings demonstrate that subtle variations in the molecular structure and interfacial charge transfer can significantly modulate the complex interplay of different spin relaxation pathways in 2D hybrid perovskites. This mechanistic insight provides a foundation for the rational design of perovskites with systemically steered spin lifetimes through the control of phonon coupling and spatial charge separation, offering new opportunities for their integration in spintronic and quantum applications.

## Conclusions

In summary, we systematically investigated spin relaxation dynamics in type-I and type-II 2D perovskites across a broad range of temperatures and excitation fluences using circularly polarized pump–probe spectroscopy. By varying the organic ligands from PEA to 4Tm and 4TCNm, we demonstrated that interlayer charge separation induced by electronically active ligands can markedly shift the dominant spin relaxation pathways. Specifically, spin relaxation changes from BAP-dominated in type-I (PEA)_2_PbI_4_ to EY- and DP-dominated in type-II (4Tm)_2_PbI_4_ and (4TCNm)_2_PbI_4_, respectively, consistent with coherent phonon signatures and temperature-dependent spin relaxation rates. These mechanistic insights highlight the sensitivity of spin dynamics to subtle molecular modulations at the organic–inorganic interface and underscore the value of systematic analysis in disentangling competing relaxation pathways. Our findings establish ligand engineering as a powerful strategy for tailoring spin properties through simultaneous control of interlayer charge separation and phonon coupling, providing a rational framework for the design of next-generation 2D semiconductors in spintronic and quantum applications.

## Materials and Methods

### Materials Synthesis

The organic ligands (4TmI and 4TCNmI) were prepared according to the previous literature.^26^ PEAI was purchased from Greatcell Solar Materials. Lead iodide (PbI_2_) was purchased from TCI America. All other reagents and solvents were obtained from Sigma-Aldrich and used without further purification.

### 2D Perovskite Thin Film Preparation

Perovskite thin films were prepared on a glass substrates. Prior to use, substrates were sequentially ultrasonicated in Alconox, deionized (DI) water, acetone, and isopropanol. The substrates were then treated with UV–ozone for 10 min and transferred into a nitrogen-filled glovebox for subsequent processing. Perovskite precursor solutions were prepared by dissolving 100 mM PEAI, 4TmI, or 4TCNmI, and 50 mM PbI_2_ in anhydrous *N*,*N*-dimethylformamide (DMF) at 60 °C for 2 h, respectively. Before spin coating, all of the precursor solutions were filtered with 0.45 μm polytetrafluoroethylene (PTFE) filters. All perovskite thin films were deposited by spin coating at 2000 rpm for 60 s, followed by thermal annealing on a hot plate for 10 min. The annealing temperatures were 100 °C for (PEA)_2_PbI_4_, 200 °C for (4Tm)_2_PbI_4_, and 180 °C for (4TCNm)_2_PbI_4_ respectively. Afterward, a protective layer of poly­(methyl methacrylate) (PMMA, >80% isotactic, Sigma-Aldrich) was deposited by spin coating a 1 wt % solution in anhydrous toluene at 4000 rpm for 30 s without further annealing.

### Linear Absorption and Photoluminescence Spectroscopy

Linear absorption spectra were collected using a Shimadzu UV-2600i spectrophotometer with an ISR-2600 extended-range integrating sphere accessory. Photoluminescence (PL) measurements were acquired on a home-built spectrophotometer. The samples were excited with a 405 nm diode laser (Thorlabs LDM405), and the emitted light was collected with a pair of 2″ parabolic mirrors and fiber-coupled to a spectrograph (Andor 328i) equipped with a charge-coupled device (CCD) camera (Newton 940). The wavelength and intensity calibration of the home-built system were performed with a Ne lamp and a certified reference light source (Ocean Optics HL-3P-CAL), respectively.

### Helicity Resolved Transient Absorption Spectroscopy

Transient absorption (TA) spectra were acquired using a home-built setup. Coherent Astrella USP (8 W, 800 nm, 5 kHz) was used as the source laser. The pump beam was generated by pumping an optical parametric amplifier (Coherent OPerA Solo) with 50% of the source. A broadband probe beam (∼450–750 nm) was generated by focusing the remaining 50% into compressed Ar gas at 20 psi and attenuating the residual 800 nm with a 750 nm short-pass filter. Dispersion of the probe was corrected by a chirped mirror pair, with instrument response function shown in Section 7 of the Supporting Information. The pump–probe delay was controlled with a motorized delay stage (Newport DL325), and the pump–probe sequence was controlled by a rotary chopper at 1.25 kHz. Transmitted probe light was detected on a shot-by-shot basis by a spectrophotometer (Avantes AvaSpec-ULS2048CL-EVO) operating at 2.5 kHz. Quarter-wave plates (Thorlabs AQWP10M-580) were placed in the paths of both the pump and probe beams to control the helicity of the light. A cryostation (Montana Instruments, CryoAdvance-50) was used to control the sample temperature, ranging from room temperature to liquid helium (5 K). Additional experimental details regarding the detection scheme and statistical treatment of the data are provided in Section 7 of the Supporting Information.

## Supplementary Material



## Notes

Fits of the charge transfer dynamics, estimation of exciton binding energy, temperature-dependent transient absorption spectra, helicity resolved measurements for (PEA)_2_PbI_4_ and (4TCNm)_2_PbI_4_, temperature-dependent spin relaxation measurements, fluence-dependent spin relaxation dynamics, and temperature-dependent coherent phonon oscillations (PDF)

## References

[1] Bati A. S. R., Zhong Y. L., Burn P. L., Nazeeruddin M. K., Shaw P. E., Batmunkh M. (2023). Next-generation applications
for
integrated perovskite solar cells. Commun. Mater..

[2] Zhang C., Park N.-G. (2024). Materials and methods
for cost-effective fabrication
of perovskite photovoltaic devices. Commun.
Mater..

[3] Olasoji A. J., Park J. K., Lee H. J., Song Y., Lee D. S., Im S. H. (2025). Metal halide perovskites: a platform
for next-generation multifunctional
devices. Adv. Ind. Eng. Chem..

[4] Quan L. N., Rand B. P., Friend R. H., Mhaisalkar S. G., Lee T.-W., Sargent E. H. (2019). Perovskites for
Next-Generation Optical
Sources. Chem. Rev..

[5] Qin T., Zhang X., Liu H., Wei Y., Huang H., Xiang B., Zhang M., Wang Z., Tang Z., Xiong Q. (2025). Coherent Exciton Spin Relaxation
Dynamics and Exciton Polaron Character
in Layered Two-Dimensional Lead-Halide Perovskites. ACS Nano.

[6] Giovanni D., Chong W. K., Liu Y. Y. F., Dewi H. A., Yin T., Lekina Y., Shen Z. X., Mathews N., Gan C. K., Sum T. C. (2018). Coherent Spin and
Quasiparticle Dynamics in Solution-Processed
Layered 2D Lead Halide Perovskites. Adv. Sci..

[7] Privitera A., Righetto M., Cacialli F., Riede M. K. (2021). Perspectives of
Organic and Perovskite-Based Spintronics. Adv.
Opt. Mater..

[8] Guo L., Hu S., Gu X., Zhang R., Wang K., Yan W., Sun X. (2024). Emerging Spintronic Materials and Functionalities. Adv. Mater..

[9] Guo S., Mihalyi-Koch W., Mao Y., Li X., Bu K., Hong H., Hautzinger M. P., Luo H., Wang D., Gu J. (2024). Exciton engineering
of 2D Ruddlesden–Popper
perovskites by synergistically tuning the intra and interlayer structures. Nat. Commun..

[10] Wu G., Liang R., Zhang Z., Ge M., Xing G., Sun G. (2021). 2D Hybrid Halide Perovskites: Structure, Properties, and Applications
in Solar Cells. Small.

[11] Zhou J., Wu M. W. (2008). Spin relaxation
due to the Bir-Aronov-Pikus mechanism in intrinsic
and $p$-type GaAs quantum wells from a fully microscopic approach. Phys. Rev. B.

[12] Boross P., Dóra B., Kiss A., Simon F. (2013). A unified
theory of
spin-relaxation due to spin-orbit coupling in metals and semiconductors. Sci. Rep..

[13] Huang W., Zhou Z., Nam S. H., Chen Q., Wang J., Zeng Z., Ge C., Li Y., Wang J., Kim Y.-H., Zhai Y. (2025). Spin Lifetime in Hybrid Organic–Inorganic
Perovskites: Mechanisms, Measurements, and Prospects for Spintronic
Applications. J. Phys. Chem. Lett..

[14] Sohn J., Lee J. M., Lee H.-W. (2024). Dyakonov-Perel-like Orbital and Spin
Relaxations in Centrosymmetric Systems. Phys.
Rev. Lett..

[15] Chen X., Lu H., Li Z., Zhai Y., Ndione P. F., Berry J. J., Zhu K., Yang Y., Beard M. C. (2018). Impact of Layer Thickness on the
Charge Carrier and Spin Coherence Lifetime in Two-Dimensional Layered
Perovskite Single Crystals. ACS Energy Lett..

[16] Zeitz D. C., Cherrette V. L., Creech S. A., Li Y., Ping Y., Zhang J. Z. (2024). Ultrafast Spin Relaxation of Charge Carriers in Strongly
Quantum Confined Methylammonium Lead Bromide Perovskite Magic-Sized
Clusters. ACS Phys. Chem. Au.

[17] Huang Y., Chen C., Gong S., Hu Q., Liu J., Chen H., Mao L., Chen X. (2024). Tuning Spin-Polarized
Lifetime at High Carrier Density through Deformation Potential in
Dion–Jacobson-Phase Perovskites. J. Am.
Chem. Soc..

[18] Shrivastava M., Bodnarchuk M. I., Hazarika A., Luther J. M., Beard M. C., Kovalenko M. V., Adarsh K. V. (2020). Polaron and Spin Dynamics in Organic–Inorganic
Lead Halide Perovskite Nanocrystals. Adv. Opt.
Mater..

[19] Song M.-S., Wang H., Hu Z.-F., Zhang Y.-P., Liu T.-Y., Wang H.-Y. (2023). The Role of Polaronic States on the Spin Dynamics in
Solution-Processed Two-Dimensional Layered Perovskite with Different
Layer Thickness. Adv. Sci..

[20] Chen X., Lu H., Wang K., Zhai Y., Lunin V., Sercel P. C., Beard M. C. (2021). Tuning
Spin-Polarized Lifetime in Two-Dimensional Metal–Halide
Perovskite through Exciton Binding Energy. J.
Am. Chem. Soc..

[21] Todd S. B., Riley D. B., Binai-Motlagh A., Clegg C., Ramachandran A., March S. A., Hoffman J. M., Hill I. G., Stoumpos C. C., Kanatzidis M. G. (2019). Detection of Rashba spin splitting in 2D organic-inorganic
perovskite via precessional carrier spin relaxation. APL Mater..

[22] Yu Z.-G., Li Y. S. (2019). Unraveling the Spin Relaxation Mechanism in Hybrid Organic–Inorganic
Perovskites. J. Phys. Chem. C.

[23] Elliott R. J. (1954). Theory
of the Effect of Spin-Orbit Coupling on Magnetic Resonance in Some
Semiconductors. Phys. Rev..

[24] Song Y., Dery H. (2012). Analysis of phonon-induced
spin relaxation processes in silicon. Phys.
Rev. B.

[25] Deng S., Snaider J. M., Gao Y., Shi E., Jin L., Schaller R. D., Dou L., Huang L. (2020). Long-lived charge separation
in two-dimensional ligand-perovskite heterostructures. J. Chem. Phys..

[26] Gao Y., Shi E., Deng S., Shiring S. B., Snaider J. M., Liang C., Yuan B., Song R., Janke S. M., Liebman-Peláez A. (2019). Molecular engineering of organic–inorganic hybrid perovskites
quantum wells. Nat. Chem..

[27] Soni A., Ghosal S., Kundar M., Pati S. K., Pal S. K. (2024). Long-Lived
Interlayer Excitons in WS2/Ruddlesden–Popper Perovskite van
der Waals Heterostructures. ACS Appl. Mater.
Interfaces.

[28] Campi D., Coriasso C. (1995). Optical nonlinearities in multiple quantum wells: Generalized
Elliott formula. Phys. Rev. B.

[29] Han B., Qiu Q., Tang Y., Lian B., Liu B., Ding S., Ma S., Luo M., Wang W., Xu B., Hsu H. (2025). Manipulating
Interlayer Carrier Relaxation Dynamics in Type-II Heterostructures
of 2D Hybrid Perovskites Through Organic Spacer Engineering. Adv. Funct. Mater..

[30] Chen B., Yu R., Xing G., Wang Y., Wang W., Chen Y., Xu X., Zhao Q. (2024). Dielectric Engineering of 2D Organic–Inorganic
Hybrid Perovskites. ACS Energy Lett..

[31] Straus D. B., Kagan C. R. (2018). Electrons, Excitons, and Phonons in Two-Dimensional
Hybrid Perovskites: Connecting Structural, Optical, and Electronic
Properties. J. Phys. Chem. Lett..

[32] Babaian D., Hill D., Yu P., Guha S. (2024). Carrier relaxation
and exciton dynamics in chemical-vapor-deposited two-dimensional hybrid
halide perovskites. J. Mater. Chem. C.

[33] Chakraborty R., Sercel P. C., Qin X., Mitzi D. B., Blum V. (2024). Design of
Two-Dimensional Hybrid Perovskites with Giant Spin Splitting and Persistent
Spin Textures. J. Am. Chem. Soc..

[34] Gan Z., Cheng Y., Chen W., Loh K. P., Jia B., Wen X. (2021). Photophysics
of 2D Organic–Inorganic Hybrid Lead Halide Perovskites:
Progress, Debates, and Challenges. Adv. Sci..

[35] Lafalce E., Amerling E., Yu Z.-G., Sercel P. C., Whittaker-Brooks L., Vardeny Z. V. (2022). Rashba splitting
in organic–inorganic lead–halide
perovskites revealed through two-photon absorption spectroscopy. Nat. Commun..

[36] Zhai Y., Baniya S., Zhang C., Li J., Haney P., Sheng C.-X., Ehrenfreund E., Vardeny Z. V. (2017). Giant Rashba splitting
in 2D organic-inorganic halide perovskites measured by transient spectroscopies. Sci. Adv..

[37] Žutić I., Fabian J., Das Sarma S. (2004). Spintronics:
Fundamentals and applications. Rev. Mod. Phys..

[38] Giovanni D., Ma H., Chua J., Grätzel M., Ramesh R., Mhaisalkar S., Mathews N., Sum T. C. (2015). Highly Spin-Polarized Carrier Dynamics
and Ultralarge Photoinduced Magnetization in CH3NH3PbI3 Perovskite
Thin Films. Nano Lett..

[39] Ohno Y., Terauchi R., Adachi T., Matsukura F., Ohno H. (1999). Spin Relaxation in GaAs(110) Quantum
Wells. Phys. Rev. Lett..

[40] Xu J., Li K., Huynh U. N., Fadel M., Huang J., Sundararaman R., Vardeny V., Ping Y. (2024). How spin relaxes and dephases in
bulk halide perovskites. Nat. Commun..

[41] Wu M. W., Jiang J. H., Weng M. Q. (2010). Spin dynamics in semiconductors. Phys. Rep..

[42] Odenthal P., Talmadge W., Gundlach N., Wang R., Zhang C., Sun D., Yu Z.-G., Valy
Vardeny Z., Li Y. S. (2017). Spin-polarized exciton
quantum beating in hybrid organic–inorganic perovskites. Nat. Phys..

[43] Zhou M., Sarmiento J. S., Fei C., Zhang X., Wang H. (2020). Effect of
Composition on the Spin Relaxation of Lead Halide Perovskites. J. Phys. Chem. Lett..

[44] Bourelle S. A., Shivanna R., Camargo F. V. A., Ghosh S., Gillett A. J., Senanayak S. P., Feldmann S., Eyre L., Ashoka A., van de Goor T. W. J. (2020). How Exciton Interactions Control Spin-Depolarization
in Layered Hybrid Perovskites. Nano Lett..

[45] Seyitliyev D., Qin X., Jana M. K., Janke S. M., Zhong X., You W., Mitzi D. B., Blum V., Gundogdu K. (2023). Coherent Phonon-Induced
Modulation of Charge Transfer in 2D Hybrid Perovskites. Adv. Funct. Mater..

[46] Fu J., Li M., Solanki A., Xu Q., Lekina Y., Ramesh S., Shen Z. X., Sum T. C. (2021). Electronic
States Modulation by Coherent
Optical Phonons in 2D Halide Perovskites. Adv.
Mater..

[47] Maity P., Yin J., Cheng B., He J.-H., Bakr O. M., Mohammed O. F. (2019). Layer-Dependent
Coherent Acoustic Phonons in Two-Dimensional Ruddlesden–Popper
Perovskite Crystals. J. Phys. Chem. Lett..

[48] Thouin F., Valverde-Chávez D. A., Quarti C., Cortecchia D., Bargigia I., Beljonne D., Petrozza A., Silva C., Srimath Kandada A. R. (2019). Phonon coherences reveal the polaronic character of
excitons in two-dimensional lead halide perovskites. Nat. Mater..

