# Supplementary material for: Mechanistic Insight into Tunable Spin Relaxation in Two-Dimensional Type-II Ligand-Perovskite Heterostructures
Source: J Am Chem Soc. 2025 Oct 29;147(45):41845–54. doi: 10.1021/jacs.5c14647 (PMC12616697; doi:10.1021/jacs.5c14647)
Supplement: Supplementary file 1 [file ja5c14647_si_001.pdf]

Supporting Information

Mechanistic Insight into Tunable Spin Relaxation in Two-Dimensional Type-II Ligand-Perovskite Heterostructures

Ashish Soni[1], Cheng Yang[1], Yu-Ting Yang[1,2], Chenjian Lin[1,2], Letian Dou[1,2], Lili Wang[1]*

[1]Department of Chemistry, Emory University, Atlanta, Georgia 30322, United States

[2]Davidson School of Chemical Engineering, Purdue University, West Lafayette, Indiana 47907, United States

*Corresponding author E-mail: lili.wang@emory.edu

Table S1. Fitting parameters for the decay kinetics at the excitonic transitions of all three samples

Sample	τ_1(ps)	τ_2(ps)	τ_3(ps)
$(PEA)_2PbI_4$	2.45 ± 0.86 (20%)	66.45 ± 39.72 (80%)	---
$(4Tm)_2PbI_4$	0.23 ± 0.02 (34%)	13.60 ± 1.52 (46%)	>500 (20%)
$(4TCNm)_2PbI_4$	0.17 ± 0.02 (41%)	17.11 ± 1.33 (42%)	>500 (17%)

1. Estimation of Exciton Binding Energy Using Generalized Elliot's Formula.

A generalized Elliot's formula was suggested by Lefebvre et al.[1] to treat the Wannier-Mott excitons confined in semiconductor superlattices and quantum wells. This is a versatile approach to account for any anisotropy of a given medium. The generalized Elliot's formula has been reported for 2D-perovskite as:[1-3]

$$\boldsymbol{\alpha(E) = \alpha_{exc.}(E) + \alpha_{cont.}(E)} \quad \text{S1}$$

$$\boldsymbol{\alpha_{exc}(E)} = A\sum_{n=1}^{\infty}\frac{Ry^{*}\Gamma(n+\alpha-2)}{(n-1)!\left[n+\frac{\alpha-3}{2}\right]^{\alpha+1}}\delta(\hbar\omega - E_n) \quad \text{S2}$$

$$\boldsymbol{\alpha_{cont.}(E)} = B\frac{\left|\Gamma\left[\frac{\alpha-1}{2}+i\gamma\right]\right|^{2}e^{\pi\gamma}\gamma^{2-\alpha}}{2^{\alpha}\pi^{2-\frac{\alpha}{2}}\Gamma(\alpha/2)}Y(\hbar\omega) \quad \text{S3}$$

The First term in equation S2 represents the excitonic contribution. Ry^* is the effective Rydberg energy corresponding to the exciton binding energy, n is the order of the excitonic peak, E_n is the energy of nth excitonic peak. The parameter $\gamma = \left(\frac{Ry^*}{E}\right)^2$, and $\delta(\hbar\omega - E_n)$ is Dirac delta function.

The second term in equation S3 represents the continuum contribution with $Y(\hbar\omega)$, the Heavyside step function, which is broadened by $\frac{1}{1+e^{-2b(\hbar\omega)}}$. The linear absorption spectra of $(PEA)_2PbI_4$, $(4Tm)_2PbI_4$, and $(4TCNm)_2PbI_4$ were modeled with equation S2, as shown in **figure S1.** The excitonic energy (E_B) is related to Rydberg energy as- $E_B = (-Ry^*)/[n + (\alpha - 3)/2]^2$. The binding energy was found to be higher for Type-I perovskite system compared to the Type-II samples.

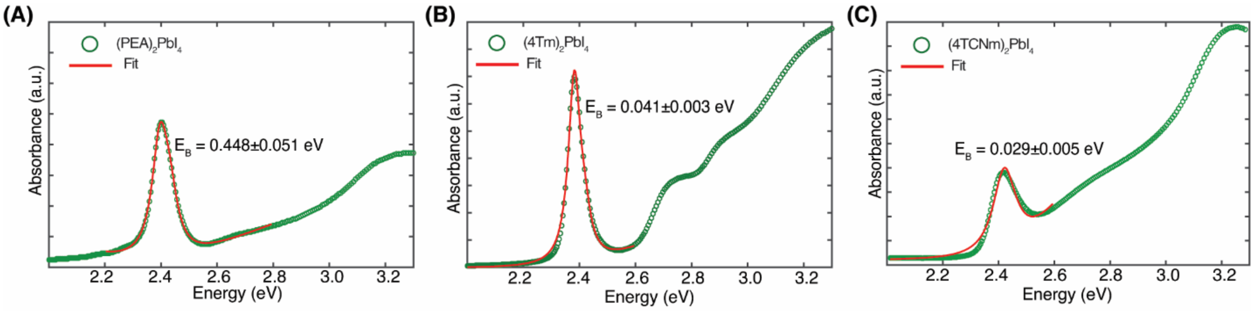

Figure S1. Absorption spectra of (A) $(PEA)_2PbI_4$, (B) $(4Tm)_2PbI_4$, and (C) $(4TCNm)_2PbI_4$. Red lines show the fitting with Elliot's formula to obtain the exciton binding energies.

2. Temperature Dependent Linear Polarized TA Spectra.

The normalized, linearly polarized TA spectra at 0.1 ps time delay for $(PEA)_2PbI_4$, $(4Tm)_2PbI_4$, and $(4TCNm)_2PbI_4$, recorded over the temperature range of 293 K to 5 K, are shown in **Figure S2**. In all three samples, the excitonic bleach feature exhibits a gradual red shift upon cooling. The magnitude of the shift varies between samples, with $(PEA)_2PbI_4$ showing the largest spectral shift, indicating stronger sensitivity of its band gap to lattice contraction and/or electron–phonon coupling. The linewidths of the excitonic bleach narrow as temperature decreases, consistent with reduced homogeneous broadening due to diminished exciton–phonon scattering. $(PEA)_2PbI_4$ and $(4Tm)_2PbI_4$ exhibit more pronounced linewidth narrowing, suggesting the presence of phonon scattering channels in these samples. Whereas $(4TCNm)_2PbI_4$ maintains a relatively broad linewidth and shows a slight blue shift upon cooling. This behavior suggests that thermal expansion and static lattice distortions dominate over exciton–phonon coupling in this system[4-6]. Among the three systems studied, $(4TCNm)_2PbI_4$ appears to exhibit the weakest exciton–phonon coupling but the strongest lattice distortion, likely due to the bulky cyano-functionalized ligand on the aromatic backbone. This structural feature, as reported in previous studies [5, 7], may contribute to the modest blue-shift observed upon cooling, distinguishing its behavior from that of the other two samples. These observations highlight the role of ligands in modulating exciton–phonon interactions, thereby influencing the temperature-dependent optical response.

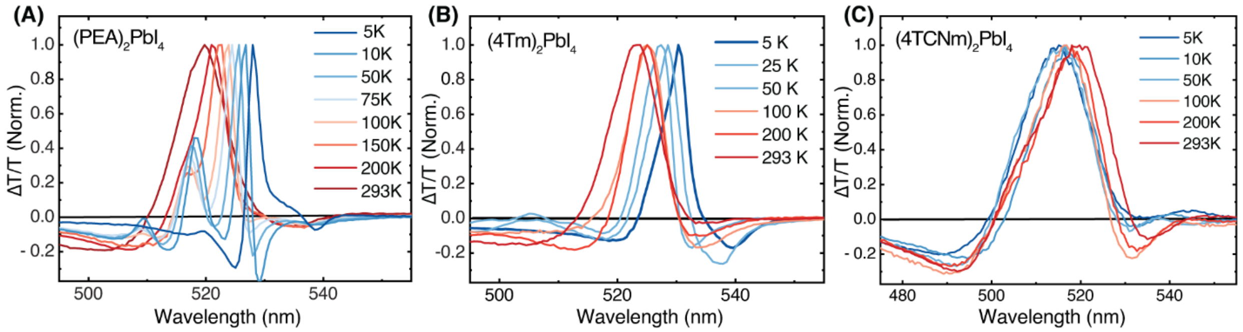

Figure S2. Normalized transient absorption spectra of (A) $(PEA)_2PbI_4$, (B) $(4Tm)_2PbI_4$, and (C) $(4TCNm)_2PbI_4$ measured over the temperature range of 5-293 K.

3. Helicity Resolved TA measurements.

The TA spectra of $(PEA)_2PbI_4$ and $(4TCNm)_2PbI_4$ at 0.1 ps time delay, measured using circularly polarized pump and probe with an excitation fluence of 3.5 $\mu J/cm^2$, are shown in **Figure S3**. The photoinduced absorption (PIA) around 530-535 nm represents the imbalance of spin polarization.

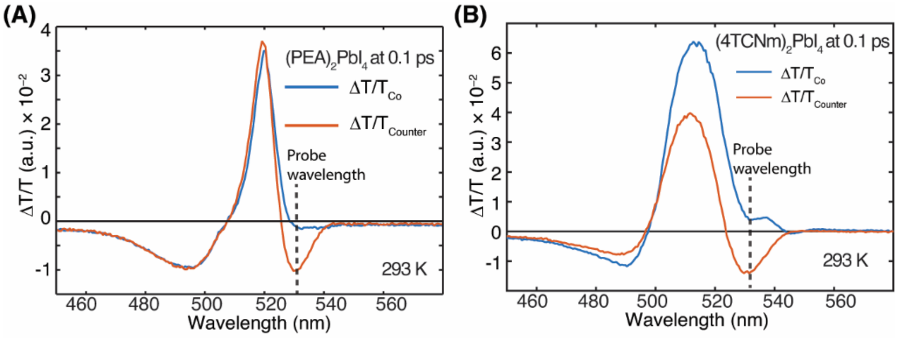

Figure S3. TA spectra of at 0.1 ps time delay for (A) $(4Tm)_2PbI_4$ and (B) $(4TCNm)_2PbI_4$, measured using co- and counter-circularly polarized pump-probe configurations.

The comparison of decay kinetics at PIA is plotted in **Figure S4,** with co-circularly polarized signals ($\Delta T/T_{Co}$, blue) and counter-circularly polarized signals ($\Delta T/T_{Counter}$, red). The difference signal ($\Delta T/T_{Co} - \Delta T/T_{Counter}$, green circles) represents the spin relaxation and is fitted with exponential function:

$$\frac{\Delta T}{T} = \sum_{i=1}^{n} A_i \exp\left(-\frac{t}{\tau_i}\right) + B \qquad \text{S4}$$

Where A_i is the amplitude, τ_i is the spin relaxation time constant, and B is a constant.

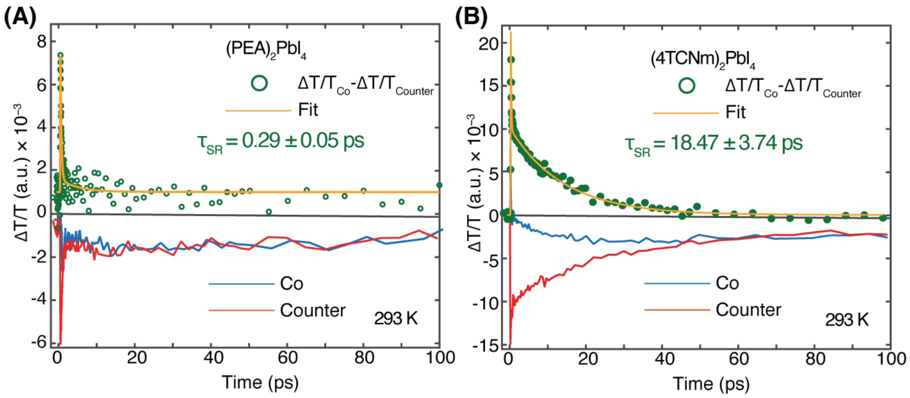

Figure S4. TA kinetics for co-circular and counter-circular polarized pump-probe of (A) $(PEA)_2PbI_4$ and $(4TCNm)_2PbI_4$. The difference in kinetics ($\Delta T/T_{Co} - \Delta T/T_{Counter}$) is plotted to show the spin relaxation and fitted with exponential function to find the spin relaxation time (τ_{SR}) at 293 K

Spin relaxation for $(PEA)_2PbI_4$ is fitted with a single exponential function (**Figure S4A),** yielding a spin relaxation time of ~0.29 ps. In contrast, spin relaxation in $(4TCNm)_2PbI_4$ requires a

biexponential fit. (**Figure S4B**). We attribute this behavior to the coexistence of exciton populations in $(4TCNm)_2PbI_4$ that exhibit distinct spin relaxation dynamics. This interpretation is supported by the distinct photoluminescence (PL) decay dynamics of $(4TCNm)_2PbI_4$ and $(4Tm)_2PbI_4$ observed in previous studies[7, 8]. $(4Tm)_2PbI_4$ exhibits a single-exponential PL decay, indicative of efficient charge transfer quenching. In contrast, $(4TCNm)_2PbI_4$ exhibits a pronounced biexponential decay, characterized by a fast component due to charge transfer and a slower radiative component. These results suggest two exciton populations in $(4TCNm)_2PbI_4$: (i) a well charge-separated population responsible for fast quenching, and (ii) a less-separated population that can still radiatively recombine. This mixed population behavior in $(4TCNm)_2PbI_4$ likely arises from the larger static lattice distortion of $(4TCNm)_2PbI_4$, attributed to its bulky ligands containing two cyano groups on the backbone.[7]

The spatial separation between the electron and hole wavefunctions is essential for suppressing exchange-driven BAP spin relaxation and extending the spin lifetimes. Accordingly, in $(4TCNm)_2PbI_4$, the fast spin relaxation component arises from trapped excitons or populations with ineffective charge separation, yielding sub-picosecond spin relaxation similar to that observed in type-I systems. The slower component corresponds to populations with effective charge separation; this component was therefore fitted and used for comparison with the other two samples.

4. Temperature Dependent Spin-Relaxation measurements.

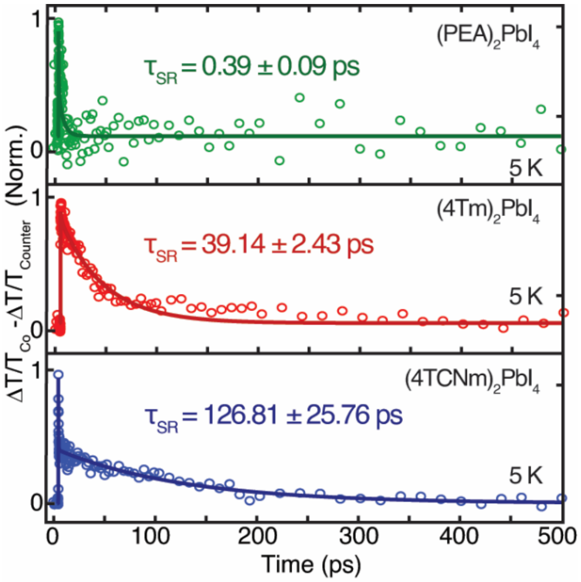

Figure S5. Normalized transient difference kinetics ($\Delta T/T_{Co} - \Delta T/T_{Counter}$) for $(PEA)_2PbI_4$, $(4Tm)_2PbI_4$, and $(4TCNm)_2PbI_4$ measured at 5 K. Solid lines represent exponential fits used to extract the relaxation time (τ_{SR}).

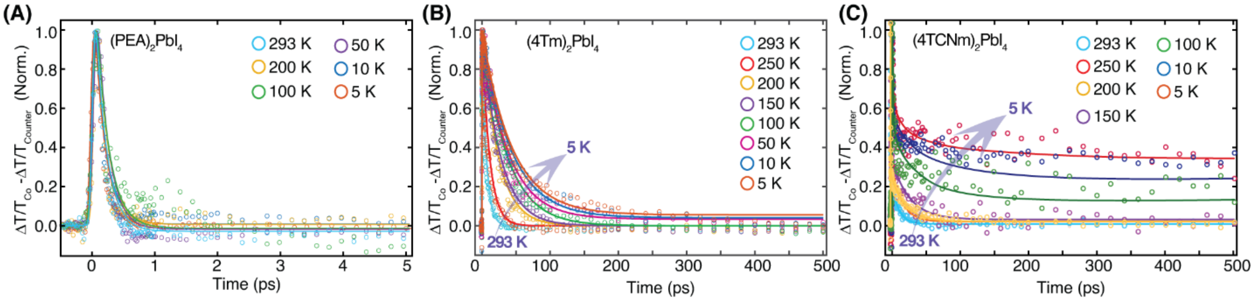

Figure S6 Spin relaxation dynamics ($\Delta T/T_{Co} - \Delta T/T_{Counter}$) for (A) $(PEA)_2PbI_4$, (B) $(4Tm)_2PbI_4$, (C) $(4TCNm)_2PbI_4$ at an excitation fluence of 3.5 $\mu J/cm^2$ and different temperatures.

5. Fluence Dependent Spin-Relaxation Dynamics

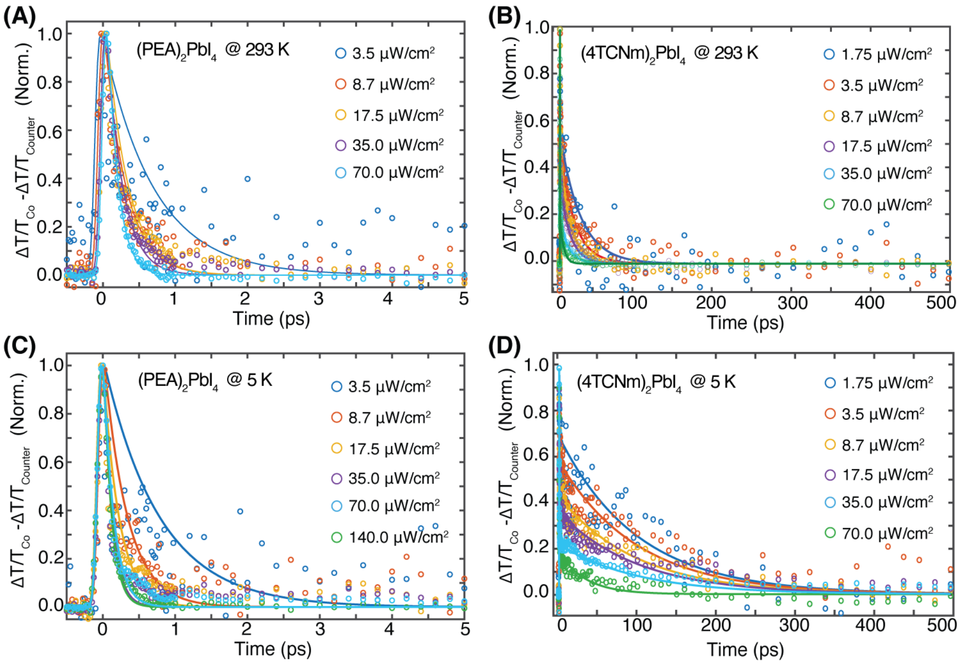

Figure S7. Normalized excitation-fluence-dependent spin relaxation dynamics at 293 K and 5 K for (A, C) $(PEA)_2PbI_4$ and (B, D) $(4Tm)_2PbI_4$. $(PEA)_2PbI_4$ and $(4TCNm)_2PbI_4$ exhibit a clear fluence dependence at both the temperatures, whereas $(4Tm)_2PbI_4$ shows no fluence dependence at 5 K, as discussed in the main text (Figure 3D).

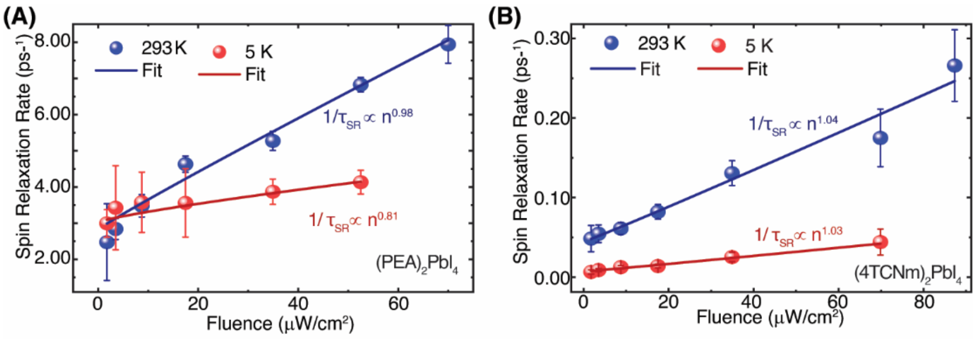

Figure S8. Fluence-dependent spin relaxation rate at 293 K and 5 K for (A) $(PEA)_2PbI_4$ and (B) $(4TCNm)_2PbI_4$. Solid lines show fits of the spin relation rate with excitation fluence.

6. Temperature Dependent Coherent Phonon Oscillations.

Coherent phonon oscillations (CPO) were analyzed for $(PEA)_2Pb_4$ and $(4Tm)_2PbI_4$ at different temperatures in the range of 5–150 K under a linearly polarized pump–probe configuration. CPO signal was extracted from TA kinetics by removing the exponential decay background. The resulting oscillation traces were fitted using a damped cosine function,

$$I(t) = \sum_{n=1}^{i} A_i \cos(2\pi f_i (t - t_0) + \phi_i)\, e^{-t/\tau_i} \qquad \text{S5}$$

In both samples, the oscillation frequency exhibits minimal variation with temperature, indicating that the underlying lattice vibrational mode is weakly temperature-dependent in this range. However, the damping rate increases gradually with temperature, suggesting enhanced phonon-phonon scattering at elevated temperatures. These observations align with the presence of CPO in both systems.

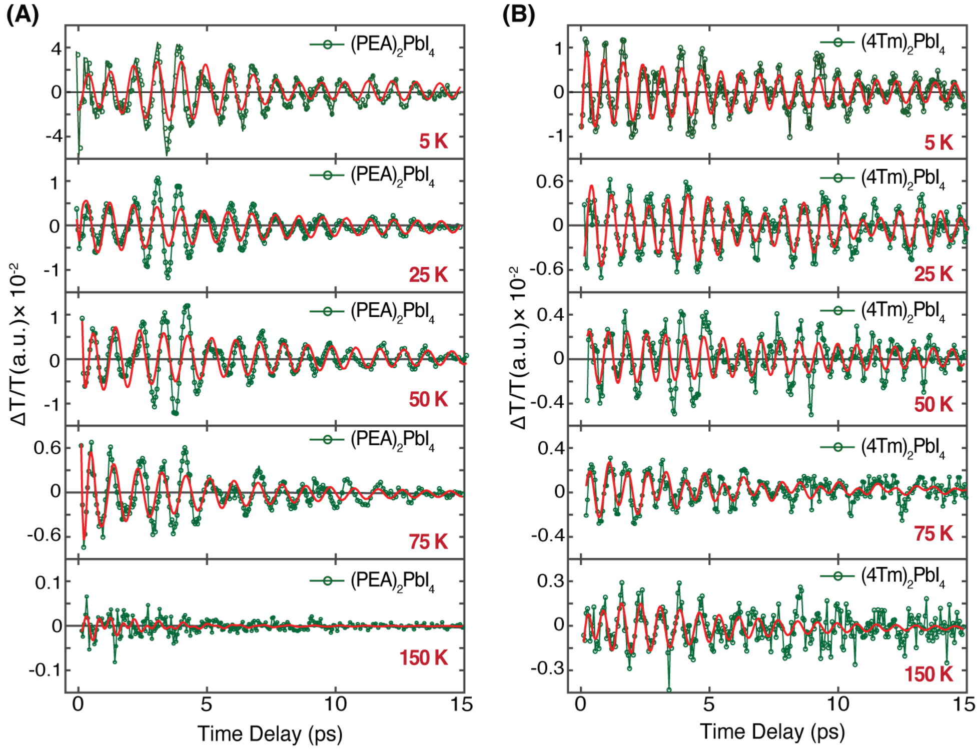

Figure S9. Temperature-dependent coherent phonon oscillations extracted by subtracting the exponential component from decay dynamics for (A) $(PEA)_2PbI_4$ and (B) $(4Tm)_2PbI_4$.

7. Additional Experimental details.

Pump–Probe and Instrument Response Function (IRF) Characterization. The spectra of the pump and probe beams, as well as the instrument response function (~ 60 fs) of our TA setup, are shown in **Figure S10**.

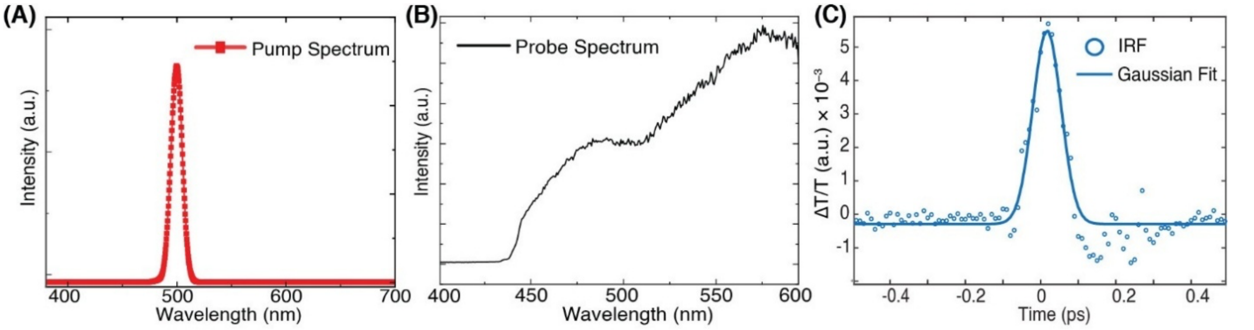

Figure S10. (A) Pump spectrum centered at 500 nm. (B) Probe spectrum covering 450–600 nm. (C) Instrument response function (IRF) measured by placing a blank sample at the pump–probe overlap. The resulting signal was fitted with a Gaussian function, yielding an IRF FWHM of ~60 fs.

Experimental robustness. To conduct the helicity resolved TA spectroscopy we employed achromatic zero-order quarter-wave plates (Thorlabs AQWP10M-580, 350–850 nm), specifically chosen to avoid any wavelength-dependent retardance. Transmitted probe light was detected on a shot-by-shot basis by a spectrophotometer (Avantes AvaSpec-ULS2048CL-EVO). In our setup, no polarization optics are placed after the sample, and the spectrometer records the total transmitted probe spectrum. Because the pump-on and pump-off signals are recorded on a shot-by-shot basis and the $\Delta T/T$ signal is computed as the transient absorption response, any static polarization dependence in detector sensitivity or grating efficiency cancels out during this normalization.

Moreover, the TA spectra measured under co- and counter-circular polarization configurations (**Figure S11**) exhibit distinct spectral differences at 0.1 ps (before significant spin depolarization) but become indistinguishable at later delay times (100 ps), once spin populations reach equilibrium. This further confirms that our observation arises from the temporal evolution of spin relaxation.

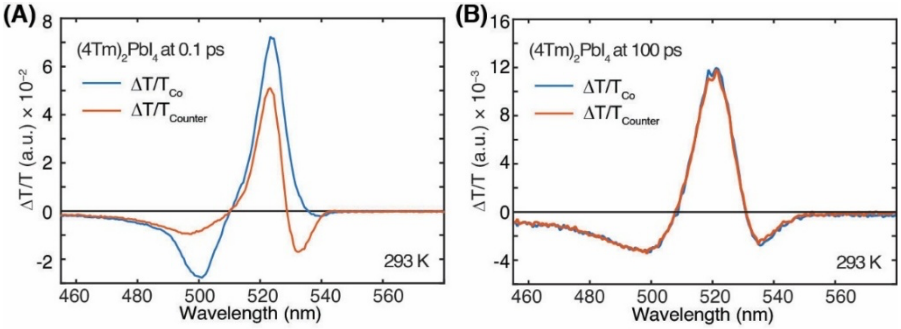

Figure S11 TA spectra of $(4Tm)_2PbI_4$ at 293 K under co-circular (blue) and counter-circular (red) pump–probe configurations, recorded at (A) 0.1 ps and (B) 100 ps time delays.

Averaging and statistical treatment of the data. The transient absorption spectra and kinetics presented in the manuscript are averaged over multiple scans to ensure reproducibility and minimize noise. Each TA measurement at a given time delay was averaged over approximately 1000 laser shots and repeated for 10–15 independent scans. In addition, each sample was measured at multiple spatial locations to confirm the reproducibility of the observed dynamics. This procedure significantly improves signal fidelity and suppresses random noise, ensuring high precision in the extracted kinetics. To illustrate this explicitly, we provided representative plots, including TA spectra at 0.1 ps for $(4Tm)_2PbI_4$ under co- and counter-circular polarization as shown in **Figure S12 (A)**, kinetic traces and corresponding spin dynamics, with error bars shown in **Figure S12 (B)** and **(C)** respectively.

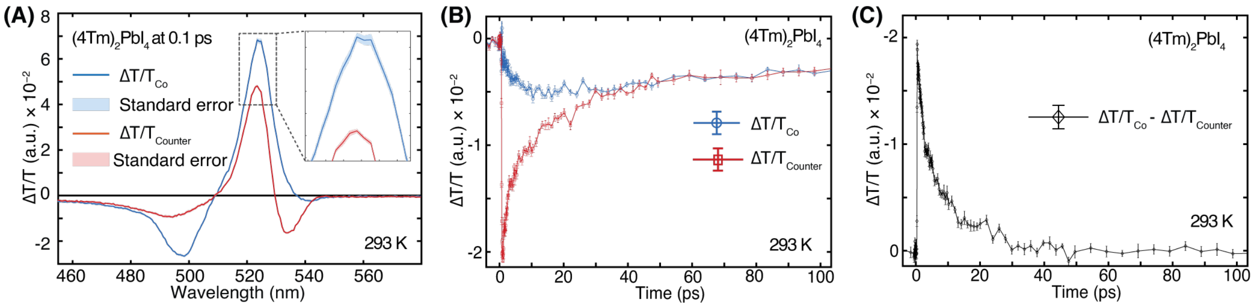

Figure S12. (A) Circularly polarized transient absorption spectra of $(4Tm)_2PbI_4$ at 0.10 ps with error bars. The error bars are not readily visible due to the high signal-to-noise ratio, but can be visualized clearly in the inset in a 10X magnified vertical scale. (B) Kinetics traces at 535 nm for co- and counter-circular configurations. (C) Spin relaxation dynamics at 535 nm obtained from the difference signal between co- and counter-circular configurations.

REFERENCES

(1) Campi, D.; Coriasso, C. Optical nonlinearities in multiple quantum wells: Generalized Elliott formula. *Physical Review B* **1995**, *51* (16), 10719-10728. DOI: 10.1103/PhysRevB.51.10719.

(2) Chen, X.; Lu, H.; Wang, K.; Zhai, Y.; Lunin, V.; Sercel, P. C.; Beard, M. C. Tuning Spin-Polarized Lifetime in Two-Dimensional Metal–Halide Perovskite through Exciton Binding Energy. *Journal of the American Chemical Society* **2021**, *143* (46), 19438-19445. DOI: 10.1021/jacs.1c08514.

(3) Babaian, D.; Hill, D.; Yu, P.; Guha, S. Carrier relaxation and exciton dynamics in chemical-vapor-deposited two-dimensional hybrid halide perovskites. *Journal of Materials Chemistry C* **2025**, *13* (1), 193-202, 10.1039/D4TC03014A. DOI: 10.1039/D4TC03014A.

(4) Zhang, Y.; Wang, R.; Li, Y.; Wang, Z.; Hu, S.; Yan, X.; Zhai, Y.; Zhang, C.; Sheng, C. Optical Properties of Two-Dimensional Perovskite Films of (C6H5C2H4NH3)2[PbI4] and (C6H5C2H4NH3)2 (CH3NH3)2[Pb3I10]. *The Journal of Physical Chemistry Letters* **2019**, *10* (1), 13-19. DOI: 10.1021/acs.jpclett.8b03458.

(5) Wang, S.; Ma, J.; Li, W.; Wang, J.; Wang, H.; Shen, H.; Li, J.; Wang, J.; Luo, H.; Li, D. Temperature-Dependent Band Gap in Two-Dimensional Perovskites: Thermal Expansion Interaction and Electron–Phonon Interaction. *The Journal of Physical Chemistry Letters* **2019**, *10* (10), 2546-2553. DOI: 10.1021/acs.jpclett.9b01011.

(6) Yu, S.; Xu, J.; Shang, X.; Ma, E.; Lin, F.; Zheng, W.; Tu, D.; Li, R.; Chen, X. Unusual Temperature Dependence of Bandgap in 2D Inorganic Lead-Halide Perovskite Nanoplatelets. *Advanced Science* **2021**, *8* (19), 2100084. DOI: https://doi.org/10.1002/advs.202100084.

(7) Gao, Y.; Shi, E.; Deng, S.; Shiring, S. B.; Snaider, J. M.; Liang, C.; Yuan, B.; Song, R.; Janke, S. M.; Liebman-Peláez, A.; et al. Molecular engineering of organic–inorganic hybrid perovskites quantum wells. *Nature Chemistry* **2019**, *11* (12), 1151-1157. DOI: 10.1038/s41557-019-0354-2.

(8) Deng, S.; Snaider, J. M.; Gao, Y.; Shi, E.; Jin, L.; Schaller, R. D.; Dou, L.; Huang, L. Long-lived charge separation in two-dimensional ligand-perovskite heterostructures. *The Journal of Chemical Physics* **2020**, *152* (4), 044711. DOI: 10.1063/1.5131801.
